# 1365. Public Health’s Role in ECHO via the Experiences of a State Department of Public Health

**DOI:** 10.1093/ofid/ofac492.1194

**Published:** 2022-12-15

**Authors:** Alexander H Molinari, Gregory S Felzien, Jackie Woodard, Suleima Salgado

**Affiliations:** Emory University, Atlanta, Georgia; Georgia Department of Public Health, Atlanta, Georgia; Georgia Department of Public Health, Atlanta, Georgia; Georgia Department of Public Health, Atlanta, Georgia

## Abstract

**Background:**

In April 2019, Georgia became the second state department of public health to implement and utilize an ECHO program to unite the Ryan White Part B HIV community. Project ECHO is a global telementoring program used to provide cutting-edge medical techniques and knowledge to medical providers in rural settings with fewer resources. Today, the GA-DPH ECHO program reaches over 300 individuals at the local, state, national, and international levels, providing essential insight to healthcare workers and a forum for discussion in several key fields, including infectious diseases and viral hepatitis.

The objective is to reveal via participation and demographical data drawn from ECHO sessions the challenges and successes in implementing and potential expanded utility of this type of program within public health, ultimately demonstrating why public health has an important role within the ECHO community.

**Image 1:**
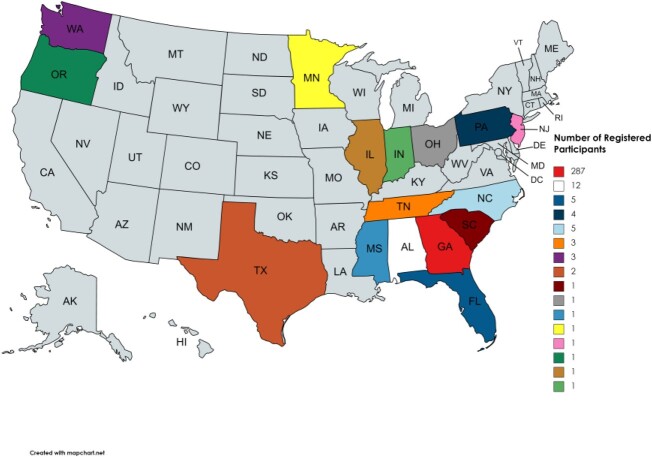
Map of United States showing outreach of GA-DPH Project ECHO. The map does not include one user based in Africa.

**Methods:**

iECHO, a partner relations management tool, was utilized in extracting demographical and participation from all GA-DPH ECHO sessions from 2019 to 2021. Data was chosen based on participation over time for each ECHO in addition to who was attending sessions while determining variations in attendance. Once compiled, data was charted into tables with iECHO software utilization to construct graphs illustrating trends over time for each ECHO to be analyzed for future expansion and evaluate the challenges.

**Results:**

The data revealed a steady growth of GA-DPH ECHO specifically the pilot infectious disease ECHO in 2019 which has grown from 14 attendees at the time to 20. A large attendance spike coincided with ECHO initiations of acute stroke and cancer registry in January 2021, with March 2021 the most attended month for the three ECHOs existing at the time. Initiated in April 2021, viral hepatitis quickly produced the most didactic sessions than any other program.

**Figure:**
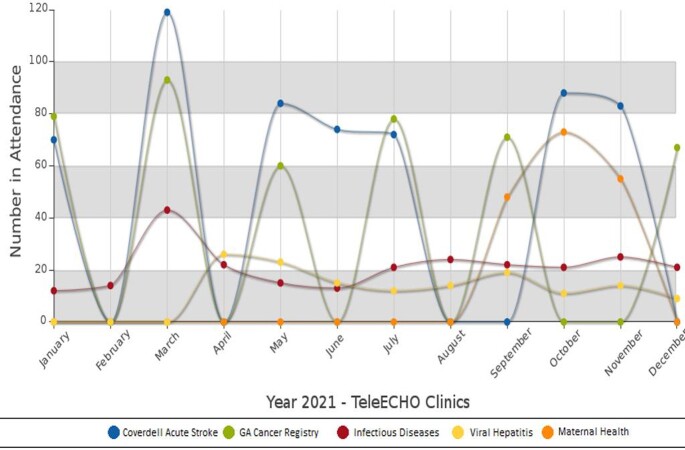
ECHO Figure 1

Graph of participation in 2021 for all five ECHOs with the highest attendance for three ECHOs during March 2021. Sessions are scheduled monthly or bi-monthly (representing months with 0 attendance) and are 1-hour in length

**Figure 2:**
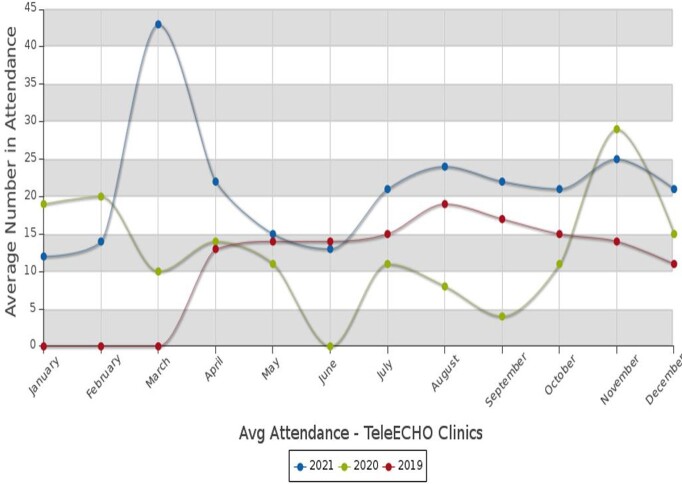
Graph of participation for Infectious Disease ECHO from 2019-2021, noted as the first GA-DPH ECHO, starting 21 months prior to other GA-DPH ECHOs

**Table 1:**
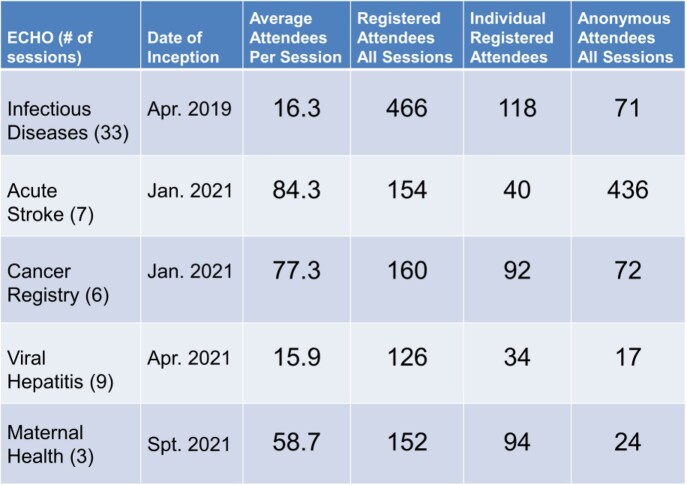
Average and total attendees for each ECHO in addition to the date of initiation and a total number of sessions. Average number of attendees include individuals registered with GA-DPH ECHO as well as anonymous (unregistered) users who join via invitation.

**Conclusion:**

GA-DPH Project ECHO has grown in attendees with a variety of sessions offered and plans for further growth and initiation of new ECHO topics. While there have been challenges, there have been far more successes with the development of 5-ECHOs. The hope is experiences of GA-DPH ECHO will show how this platform may be utilized in departments of public health across the globe.

**Disclosures:**

**All Authors**: No reported disclosures.

